# Suspended Germanium-on-Silicon Photonic Integrated Circuits Operating in the Long-Wave Infrared and Their Use for Ethanol Sensing

**DOI:** 10.1021/acsphotonics.6c00154

**Published:** 2026-04-22

**Authors:** Pen-Sheng Lin, Per-Erik Hellström, Charalampos Zervos, Frank Niklaus, Kristinn B. Gylfason

**Affiliations:** † Department of Micro and Nanosystems, School of Electrical Engineering and Computer Science, 166474 KTH Royal Institute of Technology, Stockholm SE-10044, Sweden; ‡ Department of Electronics, School of Electrical Engineering and Computer Science, 7655 KTH Royal Institute of Technology, Kista SE-16440, Sweden; § Photonics Communications Research Laboratory, 68994 National Technical University of Athens, Athens 15773, Greece

**Keywords:** optical gas sensors, ethanol gas sensors, silicon photonics, germanium waveguide, absorption spectroscopy, mid-IR waveguide

## Abstract

Germanium-based integrated photonics is gaining attention due to its potential for mid-infrared wavelength applications, including environmental sensing, industrial gas monitoring, defense, and security. However, current germanium-on-silicon platforms face significant propagation losses at wavelengths above 8 μm, and gas sensing in this regime using a germanium waveguide has not been demonstrated to date. To address this challenge, we introduce a suspended germanium-on-silicon platform, where an 11 μm deep suspension gap ensures optical mode isolation from the lossy silicon substrate. The waveguide has a low propagation loss of 3.5 dB/cm at a wavelength of 9.2 μm. Furthermore, we demonstrate on-chip ethanol gas sensing in the long-wave infrared range with a detection limit of 925 ppm using this platform. Our method paves the way for extending the operating wavelength range of germanium-on-silicon integrated photonics into the long-wave infrared.

## Graphical abstract

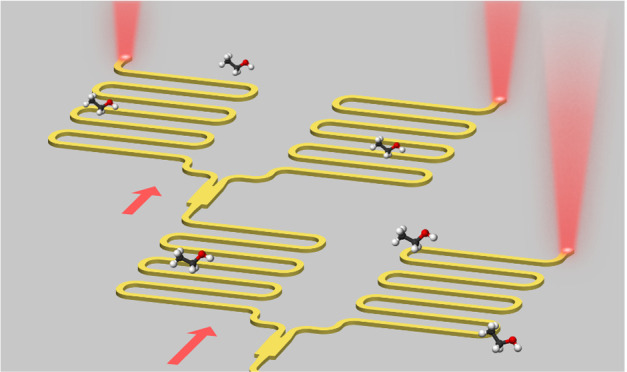

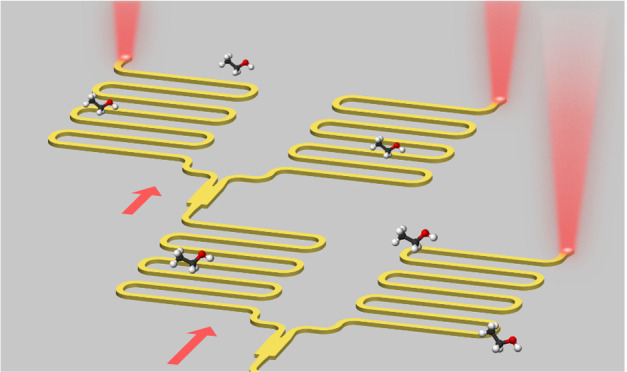

## Introduction

Integrated photonics for the mid-infrared (mid-IR, 2.5–25 μm) wavelength range has gained considerable interest recently, driven by its potential in emerging applications such as optical free-space communication^1^ active infrared imaging for medical and military uses^2^ and spectroscopic sensing.^3,4^ Within this broad mid-IR region, the long-wave infrared (LWIR, 8–14 μm) in particular is attracting growing attention for spectroscopic gas sensing because it aligns with a transmission window of atmospheric gases and provides strong, distinctive molecular absorptions that enable the detection of gases difficult to sense at shorter wavelengths. Although various mid-IR integrated photonic platforms have been demonstrated,^5,6^ most prior research has focused on silicon (Si) photonic platforms due to their compatibility with CMOS electronics fabrication and good scalability. Silicon-on-Insulator (SOI)-based Si photonic platforms have demonstrated operation at wavelengths up to 3.8 μm; however, strong light absorption in the silicon dioxide (SiO_2_) buried oxide (BOX) layer significantly increases waveguide losses beyond this wavelength.^7^ By using silicon-on-sapphire (SOS) wafers, the operating wavelength range of the Si waveguides can be extended. However, it is still limited by the transparency window of sapphire, which reaches up to approximately 5.2 μm.^8^ When using SOI Si photonic platforms, it is possible to remove the BOX layer to suspend Si waveguides. This enables the full exploitation of the mid-IR transparency of Si up to 8.5 μm.^9–11^ Removing the BOX layer also increases the waveguide surface area exposed to the surrounding environment, thereby enhancing light–matter interaction and improving the sensitivity of the waveguides in gas sensing applications. Suspended waveguides in various configurations, including metamaterial and slot waveguide designs, as well as key passive components, such as grating couplers, directional couplers, and Y-junction splitters, have been demonstrated for operation in the mid-IR spectral range.^12–15^ However, the SOI-based suspended waveguides remain insufficient to cover the long-wave infrared range beyond 8.5 μm due to the intrinsic material absorption limit of silicon.

Germanium (Ge) has emerged as a promising material for on-chip integrated mid-IR photonics due to its broad 2–14 μm transparency, high refractive index, which enables strong optical confinement and thus tight integration, and compatibility with high-volume electronics fabrication. Several Ge-based photonic platforms have been implemented in the LWIR region. Germanium-on-Silicon (GOS) platforms have achieved operating wavelengths of up to 11 μm.^16–18^ However, due to the small difference in refractive indices between Ge and Si (*n*_Ge_ = 4.0 and *n*_Si_ = 3.4 at 9.2 μm), a thick Ge device layer is required to implement waveguides, ensuring strong optical confinement and minimizing substrate losses resulting from increased radiation leaking into the high-index Si substrate at longer wavelengths. Germanium-on-insulator (GOI) and germanium-on-silicon-on-insulator (GeSOI) platforms combine the broad transparency window of Ge with the benefit of sacrificial buried layers (i.e., SiO_2_) for optical isolation.^19–21^ In GeSOI platforms, the top Ge layer is epitaxially grown on the Si device layer of an SOI wafer. In both GOI and Ge-on-SOI platforms, the BOX layer can be removed through liquid or vapor-phase hydrofluoric (HF) acid etching to avoid the strong absorption in the SiO_2_ layer. Yet, the achievable isolation gap is constrained by the thickness of the buried layer in available SOI/GOI wafers. As the wavelength increases, this limited isolation gap leads to significantly higher substrate losses. An alternative approach is Ge-rich SiGe photonic platforms that employ an epitaxially grown SiGe layer on a Si substrate, featuring a graded Ge concentration that increases from the Si substrate toward the surface of the epitaxially grown layer.^22–24^ This results in a higher refractive index near the surface, effectively confining the optical mode to the upper region of the waveguide. However, despite the high Ge content, the optical losses gradually increase at longer operating wavelengths due to multiphonon absorption in the residual Si, particularly within the LWIR range.^22^ Moreover, to prevent the optical mode from reaching the Si substrate at longer wavelengths, the SiGe layer must be sufficiently thick, typically on the order of 10 μm.

In this work, we demonstrate a suspended germanium-on-silicon (GOS) photonic platform that operates at a wavelength of 9.2 μm. To alleviate the leakage of optical power to the substrate due to the similar refractive indices of Ge and Si, we selectively remove the Si substrate beneath the waveguide, thus suspending the waveguide in air. We employ a combination of deep reactive-ion etching and tetramethylammonium hydroxide (TMAH) etching to create gaps between the waveguide and the Si substrate exceeding 10 μm, thereby minimizing substrate losses in the long-wave infrared range. Our waveguide features a channel geometry with air cladding on all sides, thereby enhancing the interaction between the evanescent field of the waveguide and the surrounding gas atmosphere containing the sensing analyte. We also demonstrate key photonic components, including an edge coupler, a grating coupler, and a multimode interference (MMI) splitter. Furthermore, we demonstrate, to our knowledge for the first time, on-chip ethanol gas sensing at a wavelength of 9.2 μm using our photonic integrated circuit. With these demonstrations, we established a foundation for a scalable mid-IR Ge integrated photonic platform.

## Results and Discussion

### Design and Fabrication

We designed a suspended Ge channel waveguide with a cross-section measuring 3.5 μm in width and 1.7 μm in height, as illustrated in Figure 1a. The suspended waveguide is supported from the bottom by Si anchors to enhance its mechanical stability (Figure 1b). Mode-solver simulations by Ansys Lumerical (2023 R2.3), incorporating literature-sourced wavelength-dependent complex refractive indices,^25^ confirm that this waveguide geometry operates over a broad optical bandwidth (2–14 μm) and transitions to single-mode operation at 6.8 μm. We extracted the propagation loss of the fundamental TE mode from simulations (Ansys Lumerical R2.4) at various suspension gaps (Figure 1c). The simulation model includes a wavelength-dependent complex refractive index to account for material losses as well as the effect of substrate leakage. The simulation begins with a 3 μm suspension gap, corresponding to the largest readily available buried oxide thickness of SOI wafers used in Si photonics. At this gap, the propagation loss grows rapidly in the LWIR region beyond an 8 μm wavelength, as the evanescent field of the guided mode increasingly overlaps with the Si substrate. The substrate losses are significantly mitigated when the waveguide suspension gap increases to 10 μm and beyond. Moreover, we also simulated the external confinement factor $\Gamma =\frac{\partial n_{\mathrm{eff}}}{\partial n_{\mathrm{clad}}}$ of our waveguide at the targeted wavelength of 9.2 μm, obtaining a value of Γ = 22%. Here, *n*_eff_ is the effective index of the guided mode and *n*_clad_ is the refractive index of the air cladding.^26^

**Figure 1:**
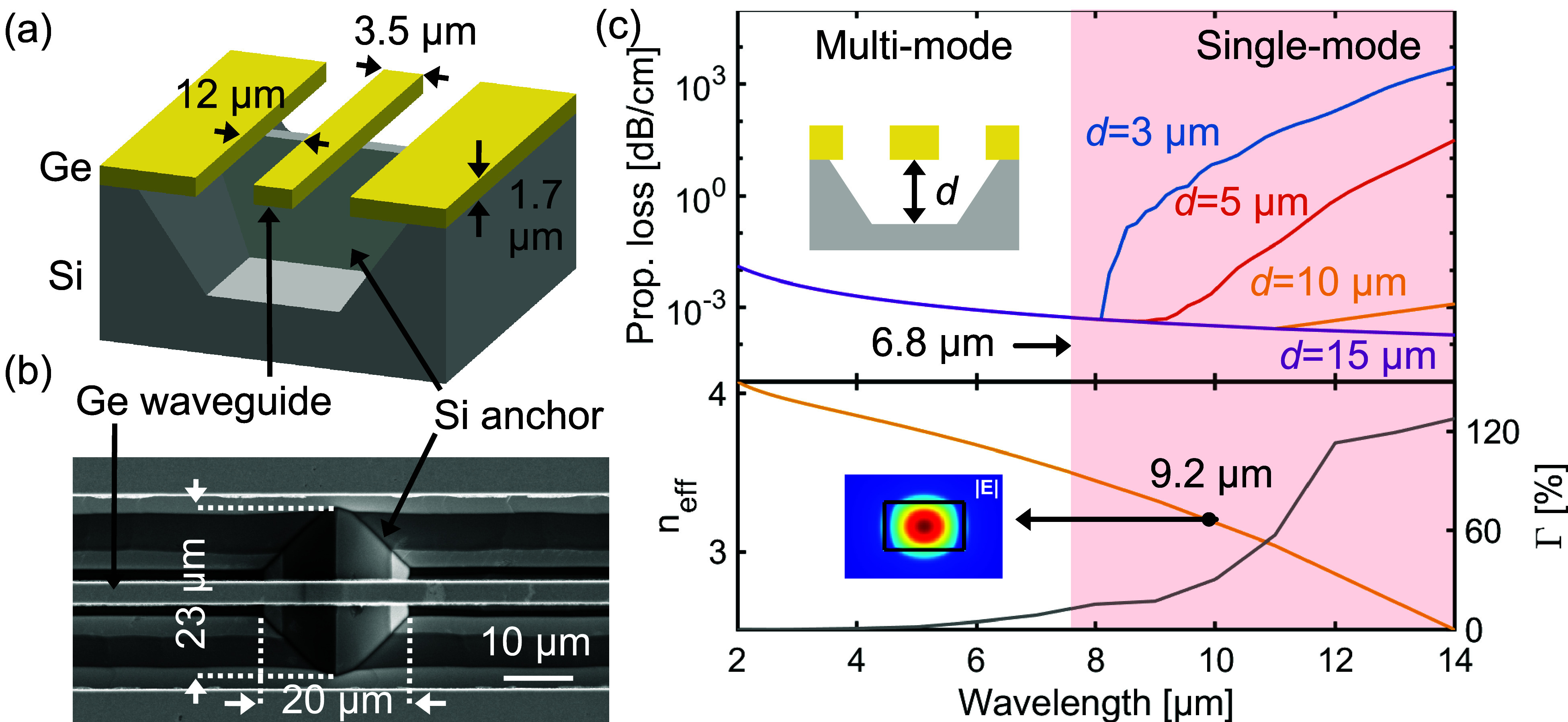
(a) Illustration of the realized suspended GOS waveguide structure with key dimensions indicated. (b) Top-view SEM image showing a suspended Ge waveguide, which is mechanically supported by an underlying trapezoidal Si anchor. The dimensions of the trapezoidal Si anchor are marked. (c) Simulation of the waveguide propagation loss due to substrate leakage for four different suspension gaps *d* (top), and of the effective index *n*_eff_ and external confinement factor Γ of the fundamental TE_00_ mode for *d* = 10 μm (bottom) across the wavelength range of interest (2–14 μm). The insets illustrate the orientation of *d* and a cross-sectional mode simulation of the TE_00_ electric field magnitude at the operating wavelength of 9.2 μm.

For characterization, we implemented a photonic integrated circuit with a splitter-tree configuration, as illustrated in the schematic overview in Figure 2a. The circuit comprises a suspended edge coupler at the input, bottom-anchored multimode interference (MMI) splitters for optical power division, and suspended grating couplers at the outputs, as shown in close-up scanning electron microscope (SEM) views in Figure 2b–d. We estimated the edge coupler and grating coupler losses to be approximately 2.76 dB/facet and 2.68 dB/coupler, respectively, based on the analysis outlined in Supporting Information Figure S1 and Figure S2. To increase the length of the waveguide in a limited area, we designed a bent waveguide with a 70 μm radius of curvature, as presented in Figure 2e. By splitting the circuit, we created different optical path lengths within the same circuit. Such a differential circuit configuration enables us to reference one circuit output to another, thereby aiding in drift correction and noise reduction.

**Figure 2:**
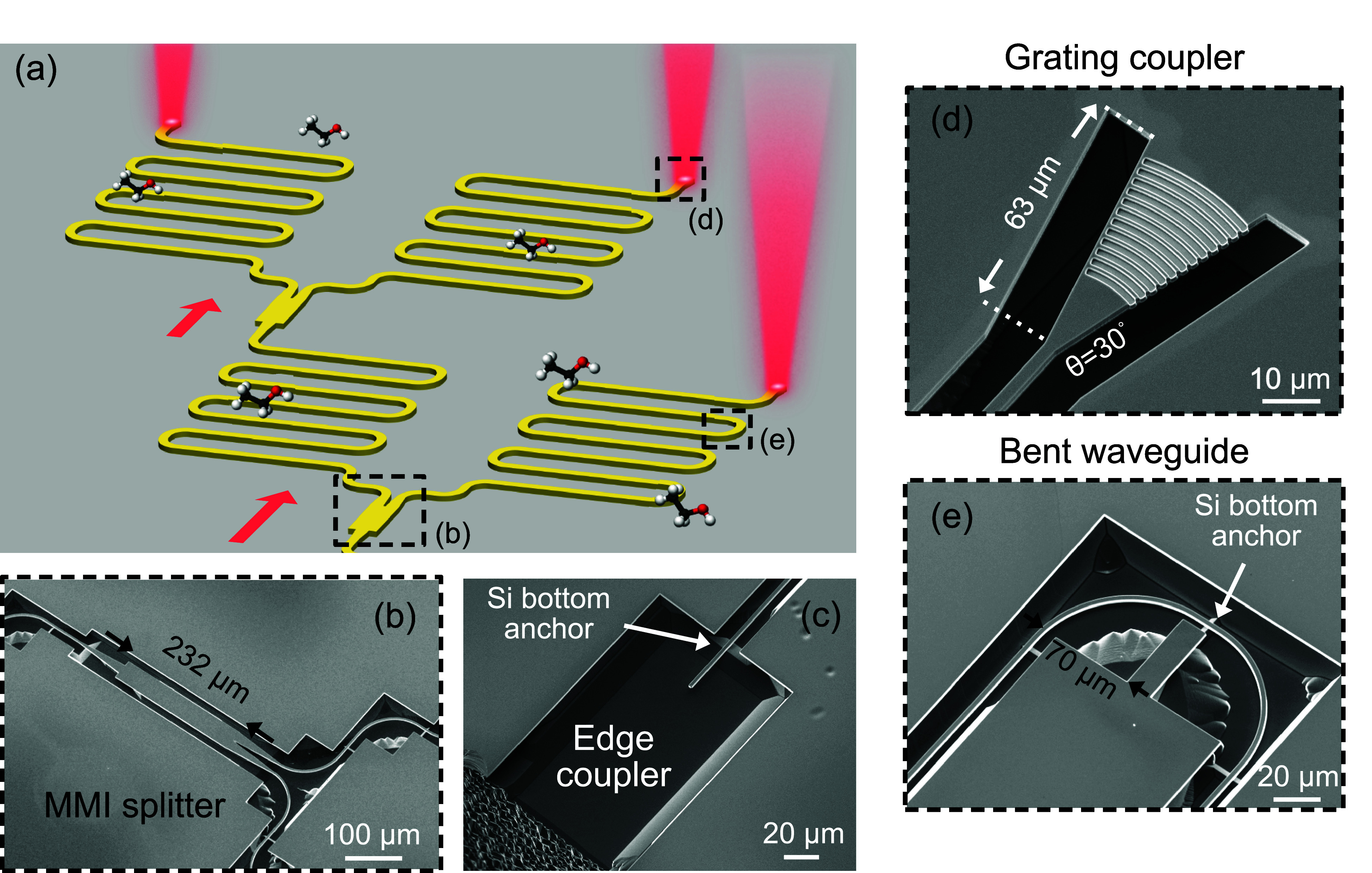
(a) Illustration of the GOS photonic integrated circuit for gas sensing, highlighting the splitter-tree configuration that enables on-chip referencing. SEM images of the essential passive components, including (b) the nonsuspended MMI splitter, (c) the edge coupler for in-coupling, (d) the grating coupler for out-coupling, and (e) the bent waveguide.

To implement our GOS photonic platform, we grew a 1.7 μm thick Ge device layer on a 100 mm diameter (100) Si wafer using an epitaxy reactor (Epsilon 2000, ASM International N.V., The Netherlands). All lithography steps in this process were performed using laser direct writing lithography (MLA 150, Heidelberg Instruments Mikrotechnik GmbH, Germany), which offers a submicrometer lithographic resolution of 500 nm. To implement the photonic integrated circuit on the GOS wafer, we first etched 650 nm deep gratings using HBr/Cl_2_ plasma reactive ion etching (P5000 PECVD, Applied Materials Inc., CA), as shown in Figure 3a **i**. Subsequently, we patterned the photonic circuit components through a second reactive ion etching step, which etched through the Ge layer and landed on the Si substrate, as illustrated in Figure 3a **ii**. In this step, the circuit was isolated from the surrounding Ge slab by a clearance of 13 μm on each side of the waveguide. This avoids light coupling to the slab at long-wave infrared wavelengths. In the third lithography step, laser direct writing lithography was performed in the exposed Si substrate regions, followed by 8 μm deep trench formation by deep reactive ion etching (DRIE), as shown in Figure 3a **iii**. Spaced 18 μm apart along the waveguides, these trenches expose a vertical facet for the subsequent anisotropic under-etching process. These trenches also dictate the depth of the suspension gap between the Ge waveguide and the Si substrate, which is crucial for minimizing substrate loss at long infrared wavelengths. In the final step, we suspended the Ge waveguides by selectively removing the Si substrate underneath the waveguide structures using anisotropic TMAH wet etching, as illustrated in Figure 3a **iv**. The etch selectivity of (100) Si to Ge is expected to exceed 1000:1, with a reported selectivity of (100) Si to Si_0.56_Ge_0.44_ being approximately 100:1.^27^ By leveraging the anisotropic etch rate of Si,^28^ the under-etch started at the deep trenches and slowed down at the (111) facets, forming trapezoidal Si prisms beneath the Ge waveguides as bottom anchors in the regions between the deep trenches, as shown in Figures 1b and 3b. The Si prism-shaped bottom anchors measure 20 μm in length and 23 μm in width, with a submicrometer-scale contact region attached to the waveguide. This allows us to create long suspended Ge waveguides without adding lateral anchors or changing the waveguide cross-section. The resulting suspension gap between the Ge waveguide and the Si substrate was 11 μm deep, as shown in Figure 3b, effectively isolating the waveguide mode from the substrate.

**Figure 3:**
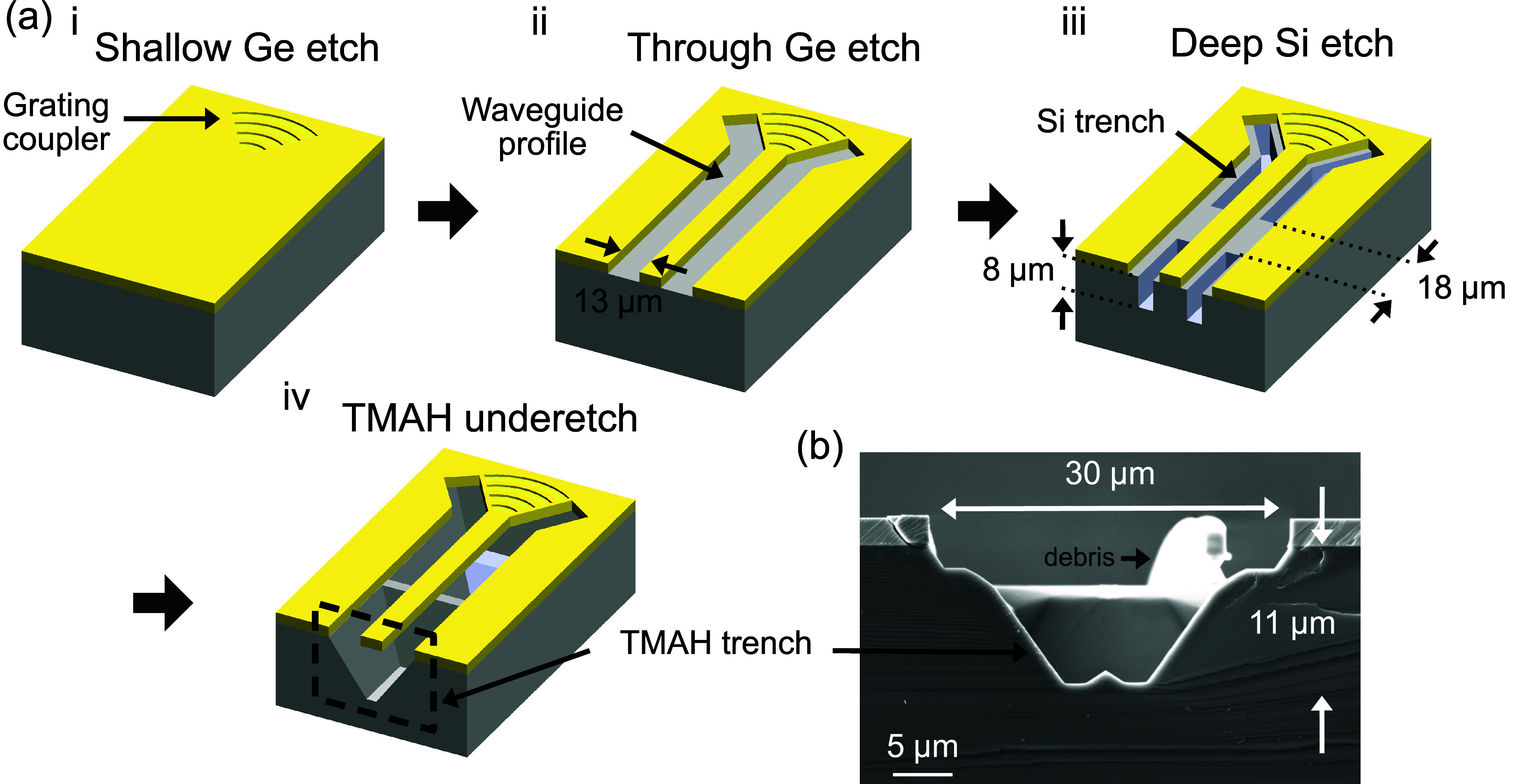
(a) Fabrication sequence of the GOS photonic integrated circuits for gas sensing. **i**. Patterning of grating couplers on the Ge device layer to define the optical output interface. **ii**. Full-depth etching through the Ge device layer to define the waveguide and circuit. **iii**.  Deep reactive-ion etching (DRIE) into the Si substrate to form the under-etch access windows. **iv**. Anisotropic TMAH wet etching of the Si substrate to suspend the GOS circuit. Trapezoidal supporting anchors form between the under-etch access windows as the etch slows at the Si (111) facets. (b) Cross-sectional SEM image of the trench formed by TMAH etching, showing an 11 μm deep suspension gap. The waveguide is absent.

### Experimental Results

To characterize our Ge photonic integrated circuit, we used the setup illustrated in Figure 4a **i**. A linearly polarized laser beam at a 9.2 μm wavelength from a quantum cascade laser (QD9500CM1, Thorlabs, USA) was focused onto the waveguide edge coupler. We used a long-wave infrared bolometric camera with a 2× microscope lens (PI640i, Optris GmbH & Co. KG, Germany) to image the chip. The camera was used both for aligning the laser and for monitoring the optical output emitted from the waveguide surface grating couplers. In addition to optical circuit characterization, the setup is also readily adaptable for nondispersive infrared (NDIR) gas sensing measurements. To conduct the NDIR ethanol gas sensing experiments, we generated ethanol vapor using a nitrogen-bubbling setup, in which N_2_ gas was passed through liquid ethanol via a flow controller (Figure 4a **i**). The generated ethanol vapor was blown over the sample using a nozzle. As a reference and to assess the circuit’s selectivity toward ethanol, we also evaluated other gases, including water vapor generated by the same bubbling method and premixed BIOGON gas (10% CO_2_, 20% O_2_, and 70% N_2_) (Linde AB, Sweden).

**Figure 4:**
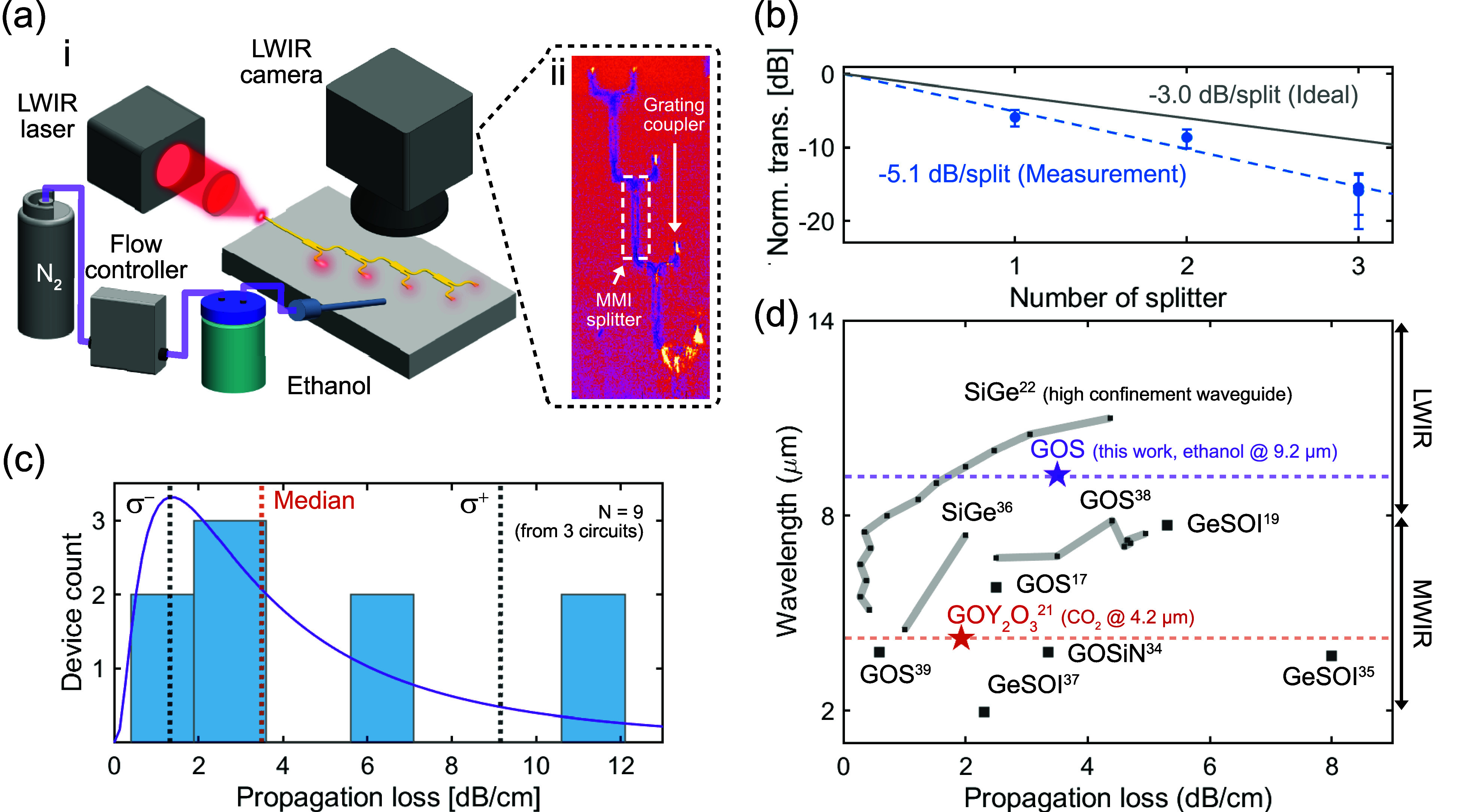
(a) **i**. Illustration of the experimental setup for optical characterization and vapor sensing. **ii**. Example image of an MMI splitter-tree circuit with four splitters under test, captured using an LWIR bolometric camera (spectral range: 8–14 μm). (b) Excess loss characterization of the MMI splitter by comparison with an ideal lossless four-stage splitter-tree circuit. The output powers at splitter stages two to four are normalized to the output power of the first stage. (c) Histogram of waveguide propagation loss with a fitted log-normal distribution. The nine data points correspond to pairwise loss measurements extracted from three three-stage splitter-tree circuits. (d) A summary of literature reports of Ge-based integrated photonic platforms, highlighting their operating wavelengths and waveguide propagation losses. ■ indicates studies reporting only waveguide characterization; ★ indicates studies including gas sensing demonstrations.

We first assessed the performance of the MMI splitter, which is required for the calibration of splitter-tree circuits. Figure 4a **ii** shows an LWIR camera image of a splitter-tree circuit with four levels. The circuit connects four identical MMI splitters in series without any additional waveguide length between each splitter. We measured the output optical power at the grating couplers at each stage and normalized these values to the optical power measured at the first stage. As shown in Figure 4b, we compared the measured splitting losses to an ideal loss-free splitter and found an excess splitting loss of 2.1 dB/split. In the current MMI splitter design, the underlying Si substrate is not removed, and we expect that the loss can be further reduced by redesigning the device as a suspended splitter.

To quantify the waveguide propagation loss, we measured a three-stage splitter tree circuit that incorporates an additional waveguide length of 6.3 mm, including the 14 bends between adjacent splitters, resembling the structure illustrated in Figure 2a. We calibrated the measured output optical powers to account for the splitter attenuation, and then calculated the pairwise slopes among the three branched outputs across three samples and fitted a log-normal distribution, yielding a median propagation loss of 3.5 dB/cm with an asymmetric uncertainty of +5.7 dB/cm and −2.2 dB/cm, as presented in Figure 4c. As shown in the statistics in Figure 4c, one group of devices clusters around 2 dB/cm, reflecting the waveguide’s baseline propagation loss. This baseline is primarily determined by the waveguide design, including the anchors, the quality of the Ge material, and the sidewall roughness achievable with our plasma etching process. A second group of devices exhibits higher propagation losses, resulting from occasional high-loss outliers, such as defects introduced by particles or nonuniformities in the etching across the samples. Fitting a log-normal distribution captures this right-skewed behavior,^29–31^ and its median provides a single, meaningful number that weighs both the typical losses and the variability observed across the evaluated devices.

To characterize the waveguide gas sensing performance, we alternately exposed the waveguide to 6% ethanol vapor in nitrogen and to ambient air. We calibrated the concentration of the ethanol vapor by comparing a free-space gas cell measurement with the HITRAN database^32^ as detailed in Supporting Information Figure S3. Figure 5a  **i** presents time-resolved responses of the output power for three successive branches, corresponding to total sensing waveguide lengths of 7.0, 13.3, and 19.6 mm. The associated changes in the fitted waveguide propagation loss are presented in Figure 5a **ii**. The waveguide exhibited increased propagation loss when exposed to ethanol vapor. The gas sensing response and recovery times of the sensing system were quantified as 187 and 401 s, respectively, by analyzing a representative ethanol/air cycle, as shown in Supporting Information Figure S4. The relatively slow response is attributed to the low gas flow rate, which is required by the nitrogen bubbling method used to generate ethanol vapor at a sufficiently high concentration. However, the intrinsic waveguide response to ethanol is expected to be fast, as it is fundamentally determined by the light–matter interaction. As shown in Figure 5b, the linear fits of the output power data in air and ethanol vapor provide the corresponding propagation losses. The additional 0.3 dB/cm propagation loss due to the presence of ethanol vapor is determined from the difference in loss between the air and ethanol measurements. Additionally, because the LWIR spectral range corresponds to an atmospheric transparency window, the waveguide exhibits strong optical selectivity against common background gases present in air. To demonstrate this using our waveguide, we applied the same gas-sensing protocol with both water vapor and BIOGON (10% CO_2_, 20% O_2_, and 70% N_2_), and, as expected, neither case produced a noticeable response.

**Figure 5:**
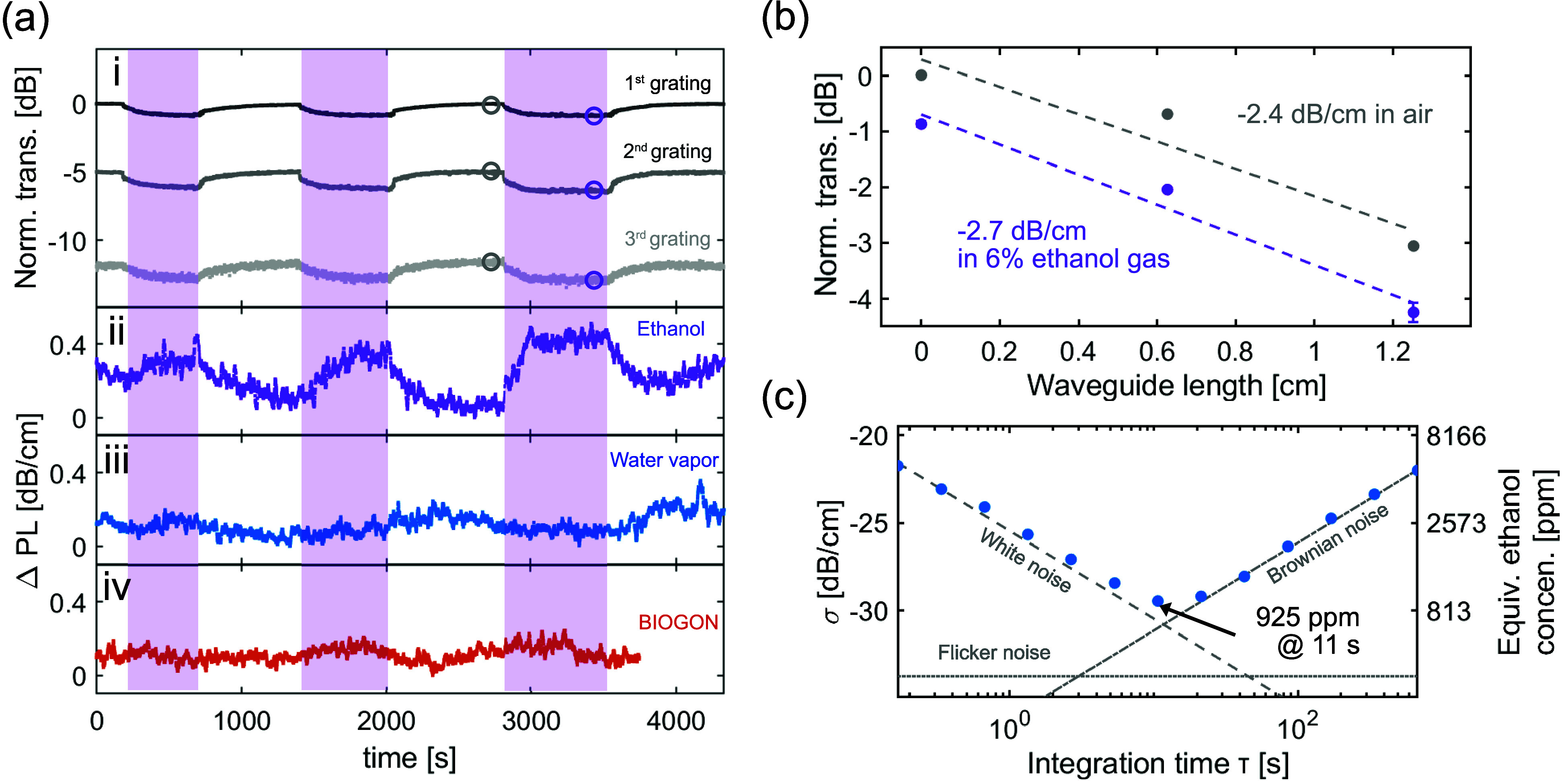
(a) **i**. Time-resolved response of the output power at the grating coupler of each branch to 6% ethanol gas in N_2_. The three successive branches correspond to sensing waveguide lengths of 7.0, 13.3, and 19.6 mm. **ii**. Temporal evolution in waveguide propagation loss (PL), calculated from the ethanol sensing response. **iii**–**iv**. Selectivity experiments using water vapor and BIOGON gas—conducted separately following the same measurement protocol as for the ethanol sensing. (b) Calculation of waveguide loss induced by ethanol absorption, obtained by comparing the fitted propagation losses in air and in 6% ethanol gas. (c) Allan deviation analysis of the time-resolved propagation loss, suggesting a noise-equivalent detection limit of 925 ppm ethanol gas at 11 s integration time.

Furthermore, to analyze the noise in the measurement and extrapolate the noise-equivalent ethanol concentration as the detection limit of our ethanol sensing system, we conducted an Allan deviation analysis with a continuous time-resolved propagation loss measurement in air over 30 min. As indicated in Figure 5c, at an integration time of 11 s, our system achieved a detection limit of 925 ppm ethanol in nitrogen. White noise dominates the Allan deviation at short integration times, while Brownian noise becomes dominant as the integration time increases.^33^ In all bolometric infrared camera measurements presented in this work, we recorded for each grating coupler a set of pixels to monitor its output power and an equal number of reference pixels. We measured the difference between the monitoring pixels and the reference pixels, which significantly reduced the contribution of flicker noise. Such a differential measurement method also mitigates the effect of the drifting temperature of the camera. Moreover, the estimated edge and grating coupler losses of 2.76 dB/facet and 2.68 dB/grating, respectively (see Supporting Information Figures S1 and S2), reduce the available optical power and consequently lower the system sensitivity. Considering these coupling losses, we believe that the suspended Ge waveguide itself has greater potential for achieving a higher signal-to-noise ratio (SNR) than demonstrated in this work. The coupling losses could be reduced through further optimization of the edge and grating couplers or by adopting waveguide-integrated light source and photodetector solutions.

Figure 4d compares our work with other Ge-based waveguide platforms reported in the literature in terms of the operation wavelengths and the waveguide propagation losses.^17,19,21,22,34–39^ Most existing works report operation wavelengths below 8 μm, limited by either the reduced optical transparency of the substrates in the LWIR region or the constraint of shallow suspension gaps that introduce significant substrate losses. Lim et al. demonstrated a Ge slot waveguide on a Y_2_O_3_ substrate with a low propagation loss of 1.9 dB/cm at a wavelength of 4.2 μm. The wide IR transparency window of the Y_2_O_3_ substrate allows the implementation of a low-confinement waveguide with an external confinement factor of 45% without the need to remove the substrate. While this enabled a limit of detection for CO_2_ gas as low as 0.6 ppm, the limited transparency of Y_2_O_3_ prevents coverage of the LWIR spectral range above 9 μm. Turpaud et al. reported a Ge-rich graded SiGe platform covering operation wavelengths from 5 to 11 μm. The thick, graded SiGe buffer layer isolates the waveguide mode from the Si substrate, allowing low propagation losses below 5 dB/cm across the entire measured range. While this approach has demonstrated low propagation losses below 2 dB/cm at wavelengths up to 10 μm, the loss increases steadily with wavelength, rising from approximately 1 dB/cm at 8 μm to 5 dB/cm at 11 μm. Turpaud et al. attributed this increased propagation loss to the increased multiphonon absorption in the residual Si at longer wavelengths. This waveguide also has a high-confinement design, which, although effective in reducing sidewall-induced scattering losses, limits its utility for evanescent-wave-based gas sensing. In contrast, our suspended Ge-on-Si waveguide incorporates an 11 μm-deep suspension trench, enabling operation at 9.2 μm. Our simulation results, shown in Figure 1c, also suggest effective waveguide mode isolation from the Si substrate throughout the 8–14 μm range. Our suspended waveguide design exposes both the top and bottom surfaces of the waveguide, which is particularly suitable for gas sensing. From the difference in propagation loss measured in air and in ethanol vapor, as shown in Figure 5b, we extracted an external confinement factor of 20% for our suspended Ge waveguide. Taken together, our suspended Ge-on-Si waveguide platform uniquely combines broad LWIR operation, low propagation loss, and substantial evanescent–field interaction, offering a spectrally versatile solution with high gas sensitivity.

## Conclusion

In summary, we have demonstrated a suspended germanium-on-silicon (GOS) integrated photonic platform incorporating an 11 μm suspension gap, which enables low-loss operation extending into the LWIR. This exceptionally large suspension gap provides sufficient optical isolation between the Ge waveguides and the Si substrate enabling the full leveraging of the intrinsic mid-IR transparency window of Ge. Our waveguides feature a low propagation loss, with a median value of 3.5 dB/cm and an asymmetric uncertainty of +5.7 dB/cm and −2.2 dB/cm at a wavelength of 9.2 μm, and demonstrated ethanol gas detection for the first time in the LWIR range, with an estimated detection limit of 925 ppm. Due to its broad spectral coverage, this platform is readily adaptable to other LWIR wavelengths and offers a promising route to scalable photonic integrated circuits that cover the full mid-IR wavelength range. These advances enable new opportunities for environmental monitoring, hyperspectral sensing, and infrared imaging technologies by supporting broadband, wavelength-agile mid-IR operation on a scalable photonic platform.

## Supplementary Material



## Notes

Figure S1: estimation of the edge coupler loss; Figure S2: simulation of the grating coupler loss; Figure S3: calibration of the ethanol vapor concentration; Figure S4: quantification of the gas sensing response time (PDF)
