# Supplementary material for: Suspended Germanium-on-Silicon Photonic Integrated Circuits Operating in the Long-Wave Infrared and Their Use for Ethanol Sensing
Source: ACS Photonics. 2026 Apr 22;13(9):2637–44. doi: 10.1021/acsphotonics.6c00154 (PMC13154358; doi:10.1021/acsphotonics.6c00154)
Supplement: Supplementary file 1 [file ph6c00154_si_001.pdf]

Suspended germanium-on-silicon photonic integrated circuits operating in the long-wave infrared for ethanol sensing

Pen-Sheng Lin,[†] Per-Erik Hellström,[‡] Charalampos Zervos,[¶] Frank Niklaus,[*,†] and Kristinn B. Gylfason[*,†]

†*Department of Micro and Nanosystems, School of Electrical Engineering and Computer Science, KTH Royal Institute of Technology, SE-10044 Stockholm, Sweden*

‡*Department of Electronics, School of Electrical Engineering and Computer Science, KTH Royal Institute of Technology, SE-16440 Kista, Sweden*

¶*Photonics Communications Research Laboratory, National Technical University of Athens, 15773 Athens, Greece*

E-mail: frank@kth.se; gylfason@kth.se

S. 1 Estimation of the edge coupler loss

We employed the Gaussian mode overlap method to estimate the input coupling loss between the focusing lens and the edge coupler. The coupling coefficient (η) is written as:

$$\eta = \left(\frac{2w_0 w}{w_0^2 + w^2} \right)^2 \quad \text{(Eq. S1)}$$

where the w_0 and w are the beam waist of the lens and the waveguide mode field radius, respectively. We used an infrared aspheric lens with a numerical aperture (NA) of 0.56 (C036TME-F, Thorlabs Inc., NJ USA) as the focusing lens. At a wavelength of 9.25 µm, the diffraction-limited Gaussian beam waist is calculated to be 5.26 µm, according to the diffraction-limited spot-size formula:

$$w_0 \approx \frac{\lambda}{\pi \mathrm{NA}} \quad \text{(Eq. S2)}$$

The waveguide mode field waist (w) was calculated to be 2.27 µm through a Mode-solver simulation by Ansys Lumerical (2023 R2.3). Therefore, in the optimal coupling condition, the maximum achievable coupling coefficient is 53 %, corresponding to a coupling loss of 2.76 dB/facet.

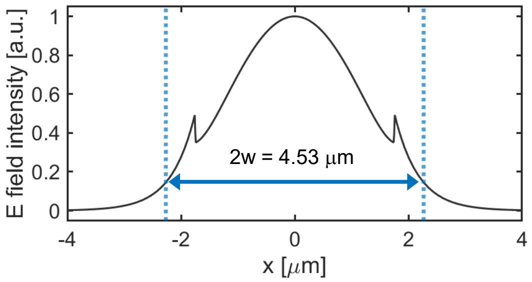

Figure S 1: Simulated waveguide mode profile with a mode diameter of 4.53 µm.

S. 2 Simulation of the grating coupler loss

We simulated the suspended grating coupler using a finite-difference-time-domain (FDTD) solver by Ansys Lumerical (2023 R2.3). We employed a parameter sweep of the grating pitch and duty cycle to identify the optimal design. The optimal grating coupler design features a pitch of 3 µm, a trench width of 1 µm, and a trench depth of 625 nm, as shown in Figure S 2a. The simulation result shows a 57 % transmission (2.68 dB/grating), a 14 % back reflection, and a transmission emission angle of 10 degrees, as presented in Figure S 2b-c.

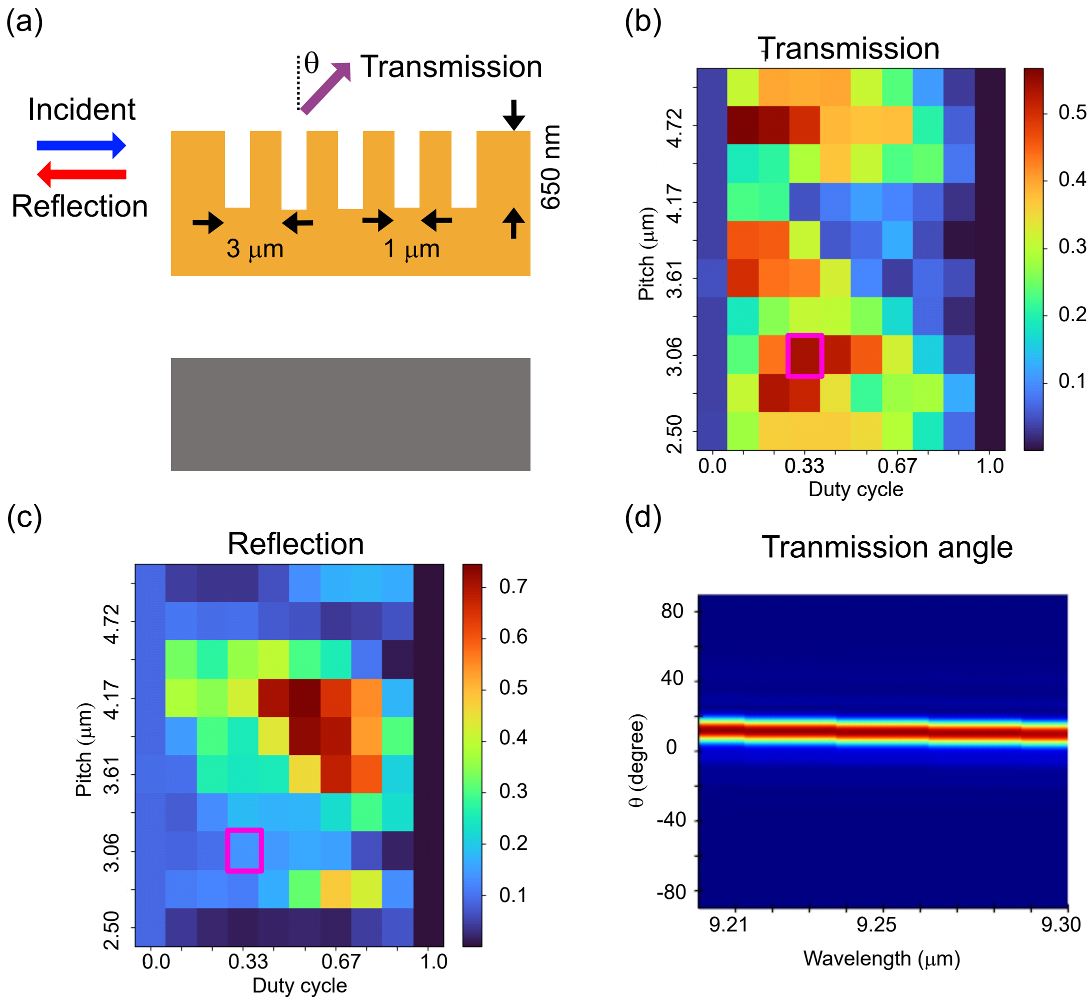

Figure S 2: Illustration and simulation of the suspended grating coupler. (a) Schematic of the grating coupler showing the grating dimensions. (b) Parameter sweep simulation results of the waveguide transmission. (c) Parameter sweep simulation results of the waveguide back reflection. (d) Simulated emission angle of the optimal grating coupler design.

S. 3 Calibration of the ethanol vapor concentration

We calibrated the concentration of the ethanol vapor which was generated using a nitrogen-bubbling technique. We performed a free-space ethanol sensing experiment using a gas cell at various wavelengths ranging from 9.24 µm to 9.28 µm. The measured wavelength-dependent ethanol absorption coefficients were compared to the reference absorption coefficients for 10 % ethanol gas from the Hitran database.

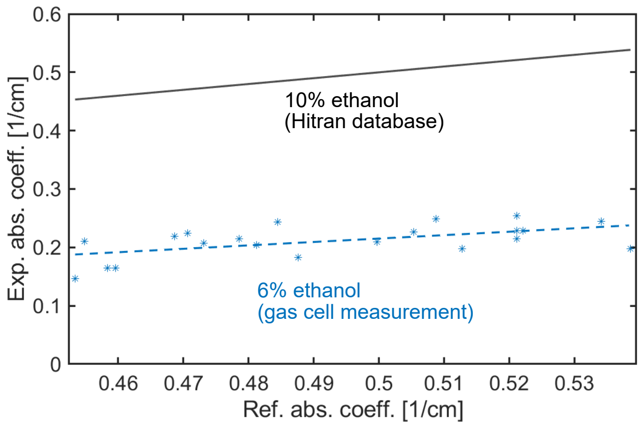

Figure S 3: Calibration of the generated ethanol vapor concentration was performed through a free-space gas cell experiment, with results validated by comparison to the HITRAN database.

S. 4 Quantification of the gas sensing response time

We quantified the gas sensing response and recovery time of the sensing system by analyzing one of the ethanol gas exposure cycles in Figure 5a **i** in the manuscript. We adopted the T_{90} metric to define the response and recovery times by determining the time points corresponding to 90 % and 10 % of the transmission decrease. The result in Figure S 3 shows a response time of 187 s and a recovery time of 401 s. The long response time is primarily limited by the low N_2 flow rate employed in the nitrogen-bubbling setup, which is necessary to generate ethanol vapor at a sufficiently high concentration for reliable detection. The

recovery process was governed by ventilation within a fume hood and therefore required a longer time than [FN9.1]ethanol detection process.

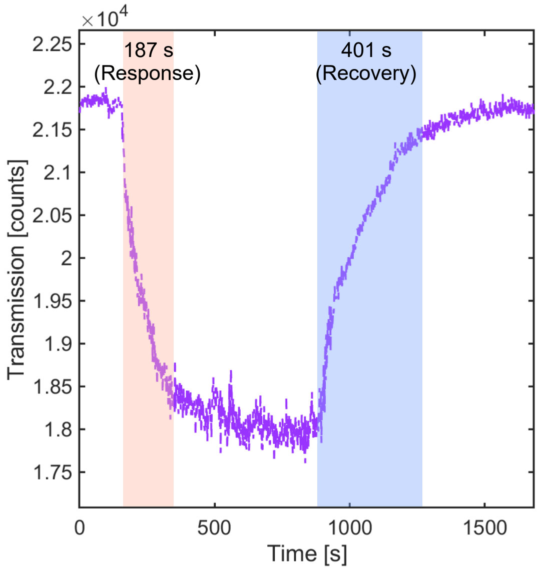

Figure S 4: Analysis of ethanol sensing response and recovery times based on waveguide transmission during an ethanol/air cycle.
